# Parenting Stress in Fathers: Do We Need Father Specific Reference Samples? And Do They Differ in Regard of Taking Parental Leave?

**DOI:** 10.3390/children9091363

**Published:** 2022-09-07

**Authors:** Nina Krüger, Johanna Nuria Rüther

**Affiliations:** 1 Department Differential Psychology and Psychological Assessment, Institute of Psychology, University of Hamburg, 20146 Hamburg, Germany; 2 Institut für Gerichtspsychologische Gutachten, 22926 Ahrensburg, Germany

**Keywords:** parenting stress, fathers, parental leave, EBI, psychometric analyses

## Abstract

The German version of the Parenting stress Index from Abidin, the Eltern-Belastungs-Inventar (EBI) merely provides reference samples of 538 mothers of children in toddlers and preschool age. Although meant to measure parenting stress, there are no father specific reference samples provided. The aim was to investigate differences in parenting stress between fathers and the provided reference samples of German mothers. Furthermore, the aim was to examine potential differences in the perceived stress between fathers who did and those who did not take parental leave. A total of 497 fathers living in Germany, of which more than half took parental leave, filled out the questionnaire via an online survey or the paper-pencil-version. All fathers completed the EBI and provided socio-economic data. The collected data were analyzed in terms of test quality, such as mean and standard deviation, corrected item–total correlation and reliability. Moreover, differences between the provided norm data and our sample were calculated. Analyses showed that fathers reported significantly higher levels of parenting stress than mothers. Furthermore, fathers taking parental leave did not differ significantly from those who did not, regarding their level of education or their perceived parenting stress. In conclusion, as it stands right now, the EBI does not adequately measure parenting stress in fathers, and father specific norms are needed to properly assess their levels of parenting stress. The results concerning parenting stress and parental leave were thus inconclusive. Furthermore, since reducing parenting stress in fathers is beneficial for the child’s development and the welfare of the parents, further studies focusing on fathers’ parenting stress are needed.

## 1. Introduction

In Western societies, historically, men played a smaller role in child rearing compared to mothers. In recent years, due to societal changes as well as public policy, the role of the father has changed, with men having greater involvement [1,2]. One of those policy changes, aimed at increasing father involvement in child development, is the introduction of paid parental leave in Germany, often including time that is held exclusively for the partner (usually the father) taking the shorter amount of parenting leave (e.g., the “Bundeselterngeld- und Elternzeitgesetz” BEEG, 2006 for Germany). 

Taking parental leave increases the amount of participation in childcare [3,4]. This effect persists even when fathers are back at work [4,5,6]. Fathers report on having a closer emotional bond with their child and on feeling more responsible for their child’s care after taking parental leave [5]. Additionally, they feel more competent and secure in raising their child [7]. One important caveat to these results seems to be the length of parenting leave taken by the father, with positive outcomes being more likely and persistent for longer parenting leave [5,8,9].

However, it is unclear whether participating in parenting leave leads to decreases in parenting stress, which has been clearly identified as one important risk factor for child development, especially during the first few years of life [10,11,12]. Further, increased parenting stress has been associated with negative, authoritarian parenting [13], attachment quality [14] and decreased social competence [10,12,15]. 

Parental stress is a specific type of stress that occurs in response to the specific demands of parenting, which in turn can be related to other types of stress within the family context, such as stress caused by social or economic factors [16]. 

Depending on the study, parenting stress has been operationalized and measured in different ways [17] Some authors assume two dimensions of parenting stress in, i.e., stressors and parenting rewards [18], some propose four factors [19]. Other authors report three dimensions of parenting stress, i.e., parental distress, parental child dysfunctional interaction and difficult child [16]. Overall, it seems common to study parental stress based on a global index (e.g., mean). 

The dimensions of parental stress are usually conceptualized as oblique, compared to other parenting dimensions such as responsiveness [20,21] and demandingness [22], which are identified as orthogonal [23]. 

The instrument most used in both clinical and research settings is the Parenting Stress Index (PSI) by Abidin [24]. The PSI is a paper-and-pencil-based questionnaire, that has been translated and adapted to many different countries, including a German version, the “Eltern Belastungs Inventar” (EBI) by Tröster [25]. The EBI consists of 48 items that assess the perceived stress of parents with babies, toddlers and pre-school aged children. It contains 12 sub-scales that belong either to the parent or child domain (see below). The EBI manual contains normative samples for the sub-scales, subsumed parent and child domains and the overall score; however, so far, the EBI has only been standardized using a sample of mothers, citing the author of the original version. They advise against using the norms for a sample of fathers, since fathers generally receive lower scores, and their stress-profile differs [26]. As for the validity of the short form of the questionnaire (PSI-SF) for fathers, a study conducted on Spanish fathers showed good validity, and they were able to largely replicate the original proposed factor structure based on data from US-American mothers [27]. Earlier validation studies, conducted on clinical samples of both fathers and mothers, found good reliability for the different subscales but were not able to replicate the proposed factor structure [28,29,30].

The research concerning gender differences in parenting stress is inconclusive. Hauenstein [31] observed higher levels of parenting stress for mothers than for fathers of chronically ill children. The same effect was found for parents of children being temporarily ill [32]. However, Krauss [33] reported similar levels of parenting stress in a sample of mothers and fathers of physically disabled children. A study on households in which both mother and father worked additionally reported similar levels of parenting stress [34]. The only difference found was that partnership satisfaction was a stronger indicator for the fathers’ amount of parenting stress. The authors concluded that the observed gender differences and role expectations are most likely determined by socialized gender-roles. Thus, if the parents voluntarily share more of the responsibilities inside and outside the household, their reactions to the stressors created by work and family life should become more similar [34].

The aim of the current study is to investigate parenting stress of German fathers and compare their data to the provided reference samples of German mothers. Furthermore, the current study will examine the psychometrics and the structure of the EBI in this sample. Additionally, the current study explores whether there are differences in the perceived stress between fathers who did and those who did not take parental leave. Due to of the exploratory quality of the data, no hypotheses were formulated to prevent data mining. The literature (see above) gives some hints that taking parental leave on the one hand could minimize perceived parental stress in fathers and on the other hand could lead to more stress in occupational contexts. Considering the missing reference samples of the EBI for fathers, we decided to explore this question and not statistically examine it. 

## 2. Materials and Methods

### 2.1. Sample

The data collection was realized within the framework of three master’s theses. Therefore, a paper-and-pencil form of the questionnaire as well as an online version using the onlinetool “unipark” (www.unipark.com, accessed on 1 September 2022) were used for data collection. Fathers with at least one child under eleven years were recruited between 2011 and 2018. For recruitment, several ways were used, such as posting the study in professional and private networks, online forums and counselling centers. The final invitation included the information that participants should be fathers with the focus on children aged 0–4. In total, more than 862 fathers were approached, of whom 497 (58%) agreed to participate in the study. The reasons for refusal were no children in the age range or no father being present in the family and no time to complete the questionnaire. On average the fathers were 37.6 years old (*SD* = 7.43). A total of 482 fathers (97%) were living in a relationship during the data collection period, and 10 fathers (2%) were single parents. On average, every father had 1.7 children (*SD* = 0.89); 255 of the youngest children were boys and 242 girls; 474 (95.4%) of the fathers were German, 21 (4.2%) from other EU-states, one (0.2%) from other states, and one data set (0.2%) was missing information on nationality. The mean weekly work time was 38.4 h (*SD* = 11.33) with a minimum of 0 and a maximum of 70 h a week. A total of 298 (60%) fathers took parental leave for at least one child, 181 (36.4%) did not, and 18 (3.6%) had missing values. The minimum of months taken was 1; the maximum 60, on average 5.8 months, 8 participants (2.7%) did not include information about the length of leave taken. In Table 1 the sociodemographic characteristics of each of the three samples as well as for the whole sample and the reference sample [25] are displayed.

The overall parenting stress score differs in dependence of the sample, although different measurements were included in each thesis (*F* = 140.252, *df* = 2, *p* = < 0.001).

### 2.2. Measurements

#### 2.2.1. Procedure

The study was conducted as a paper and pencil questionnaire as well as an online survey and included three parts. First, socio-demographic data were collected, e.g., age of the youngest child, age of the participants, family status and length of time children were in child-care outside of the family. Depending on the study version some additional closed or open questions were included, e.g., referring to role expectations or the motivation to take parental leave.

#### 2.2.2. General Information on the EBI

Next participants filled out the “Eltern-Belastungs-Inventar” (EBI), a German version of the Parenting Stress Index (PSI) from Abidin [24]. The original form of the PSI was translated by a native speaker and then evaluated in several small samples of mothers with children of nursery school or preschool age. For simplification, the items, which had different possibilities of answering, were recoded to the predominant format of answers: a 5-point Likert scale [25]. The additional scale “Defensive Responding” was removed due to a lack of empirical validation. Based on the first experiences with the translated version of the PSI, some items were removed or modified [25]. For information about reliability, validity and references see Section 2.2.3.

#### 2.2.3. Scales of the EBI

The EBI consists of 12 subscales, which are based on two different sources of parenting stress: the impact of child’s behavior and the characteristics of the parents and/or the environment. The 12 subscales are: Distractibility/Hyperactivity (Hyperaktivität/Ablenkbarkeit—HA = increased activity and distractibility of the child), Mood (Stimmung—ST = capriciousness, easy excitability and dissatisfaction of the child), Acceptability (Akzeptierbarkeit—AZ = parental disappointment that the child does not meet their expectations and requirements), Demandingness (Anforderung—AN = increased demands in the upbringing, care and care of the child) and Adaptability (Anpassungsfähigkeit—AP = difficulties of the child in aligning its behavior with the demands of everyday life) as a description of the impact of the child, operationalized as the scale “child characteristics”. One item of this scale is “I sometimes have the feeling that my child is constantly demanding on me.” (Demandingness). The following scales, Parental Attachment (Elterliche Bindung—BN = distanced relationship with the child, which is expressed in the insecurity of empathizing with the child and reliably assessing its needs), Isolation (Soziale Isolation—SI = lack of social contacts outside of the family and difficulties in maintaining social contacts outside of the family), Competence (Elterliche Kompetenz—EK = concerns not being able to cope with the demands of raising and caring for the child), Depression (Depression—DP = depressed mood, feelings of guilt and self-doubt about fulfilling responsibilities as a mother or father), Health (Gesundheit—GS = physical discomfort, physical and mental exhaustion and loss of energy), Role Restriction (Persönliche Einschränkung—PE = restrictions in the way of life and neglecting one’s own needs in favor of family obligations concerning the upbringing and care of the child) and Spouse (Partnerbeziehung—PB = impairment of the relationship with the partner due to the demands of bringing up and caring for the child) belong for the “parental characteristics”. One item of this scale is “I sometimes feel restricted by the responsibility of being a mother/a father.” (personnel restrictions). Every scale consists of four items which can be answered on a 5-point Likert scale from 1= “strongly disagree” to 5 = “strongly agree”.

#### 2.2.4. Interpretation of the EBI 

The sum score of all subscales makes up the total stress score and can be translated in standard T-values. T-values ≥ 60 indicates strong pressure whereas T-values ≥ 70 connote very strong feelings of stress in parents. In a second step, the subscales can be interpreted regarding their interconnection. This interpretation should be done carefully since the subscales are composed of only four items each. They can offer indications for supporting interventions and help to generate diagnostic hypotheses about the sources of parenting stress [25]. For example, the subscale social isolation (Soziale Isolation, SI) describes a lack of integration of the parents in a functioning social network. Sample items are “I feel trapped by parenting responsibilities” and “My child cries or fusses more often than other children.” A lack of social support can lead to disregard of the duties of education [24]. For an overview of the subscales and their meaning, see [25]. The EBI contains norms for mothers of infants and children of preschool age. The author states that the norm can be extended to mothers of older children. The reliability of the EBI is satisfying, and several studies for diploma theses have provided evidence that the EBI is a valid measure of increased self-reported parenting stress in mothers [35]. However, the EBI lacks a sufficient standardization for the assessment of parenting stress in fathers. 

### 2.3. Data Analysis

Missing data were replaced by the mean of each subscale if fewer than two items within the subscale were missing, as recommended by the test authors [25]. In total, 23 missing items were replaced by the mean of each subscale (≤0.001%). 

Cronbach’s alpha was used to examine the internal consistency of each subscale. On account of the exploratory approach, no stepwise deletion in regard of the reliability was applied. 

The validity of each item within each subscale of the EBI was assessed using psychometric item analyses [36]. Therefore, the mean, standard deviation, corrected item–total correlation was reported. Differences in scores between fathers and the reported means of the mothers were tested with a single t-test. 

Furthermore, potential differences within each subscale with regards to parental leave experiences were investigated using t-tests or if not normally distributed using Mann–Whitney–U-Tests [37]. 

The construct validity of EBI in fathers was evaluated via principal component axes analysis (PCA, [36]), using the statistical program IBM SPSS 25 and 28. The PCA is a mathematical procedure that transforms a number of (possibly) correlated variables into a (smaller) number of uncorrelated variables called principal components. Principal components analysis is similar to another multivariate procedure called Factor Analysis, which are usually used to explore scales of a new or revised measurement.

To assess potential differences in parenting stress between fathers taking parental leave and the control sample of fathers who had not taken leave, independent samples *t*-Tests were used (Greenhouse–Geissner corrected statistics were reported in cases were the was no homogeneity of variances). Furthermore, we controlled for income, education, the number of children, children’s gender, and the age of fathers. 

## 3. Results

### 3.1. Psychometric Characteristics of the EBI in Fathers

Overall, there were similar Means and Standard Deviations for each item regarding the specifications of the EBI [25]. The corrected item–total correlation showed some low connection, and two items correlated negatively or not at all with their respective scales. The first item (“My child is much more active than other children”) did not correlate with its respective Hyperactivity Scale (*r_tt_* = 0.001). The 35th item (“It depresses me when I respond irritably to my child”) was negatively correlated with its respective Depression Scale (*r_tt_* = −0.078). For detailed item characteristics see Table 2 (child subscales) and Table 3 (parent subscales).

### 3.2. Reliability of the EBI in Fathers

Overall, there were sufficient reliabilities for EBI subscales in our sample of fathers, with the exception of the Depression Scale (Cronbach’s alpha = 0.576) and the scale Spouse (Cronbach’s alpha = 0.442). However, acceptable reliabilities were reported for mothers (see Table 2). In contrast, there were substantially higher reliabilities for Mood (Cronbach’s alpha = 0.937) and Parental Attachment (Cronbach’s alpha = 0.783) for fathers compared to mothers.

### 3.3. Structure of the EBI in Fathers

An exploratory principal component analysis calculated eight factors, which did not clearly differentiate. Using the scree plot, two main factors with an Eigenvalue greater than 2 and a calculated sum square of 47.4% were found. Even after promax-rotation, most items were positively correlated to the first factor. In sum, there was no unambiguous classification of most items to one of the two factors.

### 3.4. Father Specific Reference Samples for Parenting Stress

There was a significant difference (*p* < 0.001, Cohen’s *d* = 1.259 (1.124–1.395)) between the sub-scale Child Characteristics of fathers (*M* = 58.90, *SD* = 18.559, *N* = 497) and mothers (*M* = 39.43, *SD* = 11.61, *N* = 503). The mean of the sub-scale Parental Characteristics for fathers (*M* = 82.39, *SD* = 19.633, *N* = 436) also differed significantly (*p* < 0.001, Cohen’s *d* = 1.125 (0.987–1.264)) from mothers (*M* = 60.81, *SD* = 18.64, *N* = 497). Overall, there was also a significant difference in the (summed up) Parenting Stress Index (*p* < 0.001, Cohen’s *d* = 1.285 (1.143–1.428)), with fathers having a higher mean score of 141.21 (*SD* = 35.445, *N* = 495) in comparison to mothers’ mean score of 100.23 (*SD* = 27.12, *N* = 422). Table 4 shows the descriptive characteristics of the overall score and the subscales of the current sample.

### 3.5. Comparing Fathers Taking Parenting Leave against Fathers Who Did Not Take Parenting Leave

There was no systematic effect of gender (meaning the gender of the youngest child), and thus, data were collapsed across gender. Data were normally distributed for the overall score of parenting stress (*p* > 0.05; tested with Shapiro–Wilk). 

There was no difference between fathers taking leave and fathers not taking leave concerning number of children (*t* = −0.393, *df* = 359, *p* = 0.695) or income (*U* = 26,460, *p* = 0.87). However, they did differ significantly in their levels of education with fathers taking parenting leave having a higher level of education on average (*Mdn* = 251.17) than fathers who did not take parenting leave (*Mdn* = 221.6), *U* = 23,639, *p* < 0.001. 

The overall parenting stress score was significantly correlated with fathers’ age, *r*(494) = 0.11, *p* = 0.014 and income *r_s_*(474) = 0.216, *p* < 0.001 as well as education *rs*(494) = 0.109, *p* = 0.015. Parenting stress was not affected by taking parenting leave, *t* = 0.256, *df* = 475, *p* = 0.72. This effect stayed the same when only including fathers who took parenting time longer than 3 months, *t* = −0.029, *df* = 299, *p* = 0.977.

## 4. Discussion

According to the descriptive statistics, the EBI should be capable of recording different levels of parenting stress in fathers compared to mothers. 

Regarding the corrected item–total correlation, there were two items not or negatively connected to their respective subscales. The first item of the Hyperactivity Scale (“My child is much more active than other children”) is valueless or maybe even positive connoted. The other items include more negatively associated aspects of Hyperactivity, such as “My child is unfocused.”, “My child has trouble in…” or “It is exhausting me, that my child is so active.” The first item of the Depression Scale (“It depresses me when I respond irritably to my child”) includes the aspect of feeling sorry or responsible for something going wrong in their daily lives with their child. The other items are aimed at general feelings of doing the wrong thing or not receiving enough support, such as “Because I am doing something wrong, my child is behaving bad.”, “I am depressed, when I am wrong in education…” and “It is my fault, if my child does something wrong.”.

Similar to the Depression scale, the Spouse scale shows low item–scale correlations. One reason for this might be that the provided items do not measure the intended construct. On the other hand, the scales have very low internal consistencies, presumably because of their heterogeneity, so it might be a methodological issue.

Overall, the unsatisfactory sum square of the EBI in fathers, with no clear factor structure, implies that the construct of parenting stress in fathers differs significantly from mothers. To explore this structure, a confirmatory factor analysis using a pathway analysis should be used in future research [36]). Future research should also include the theoretical conception of whether the dimensions of parental stress are assumed to be oblique or orthogonal, and this should be verified statistically for the EBI [25].

The reported significantly higher perceived parenting stress in fathers and the overall score give evidence that a separate reference sample for fathers should be provided. Alternatively, it might be that the consulted fathers are more (highly) stressed than the mothers in the reported reference samples. The questionnaire was assessed in kindergartens, primary schools and via father specific platforms on the internet. It seems likely that a representative sample of German fathers was reached. To verify this, a new study should be conducted that includes the subjective level of parenting stress of both parents to analyse the differences of fathers and mothers in a dependent sample. 

Reported parenting stress was positively correlated with fathers’ income and education. At first glance, this would seem counter intuitive, since a higher income and education should mean the presence of more resources that can be applied to childcare. Potentially, a higher income and education are related to fathers’ expectations concerning their own abilities as a father as well as their expectations of their child leading to an increase in stress due to the perceived difference between their expected parental qualities and their perceived performance as a father. 

Contrary to what might be expected from the literature on parenting leave, we did not find a difference in parenting stress for fathers taking leave and fathers not taking leave. This was also true for fathers taking longer leave (>3 months). The stressors that are important for parenting stress might not be affected by taking parenting leave, contrary to the hypothesis by Deater-Deckard [11] who proposed that conflicts regarding the sharing of tasks and responsibilities within the household are the primary factors for discontent among parents of young children. Those conflicts in turn lead to increased parenting stress. Deater-Deckard [11] speculates that fathers taking parental leave might lead to a more flexible division of household responsibilities and to an overall higher participation in childcare and household tasks. This in turn could lower parenting stress in both parents. We know taking parenting leave has been shown to have a positive effect on shared household tasks, though this was usually only true for fathers taking longer leave than the two or three months set aside specifically for fathers [3,4,38]. However, even when including only those fathers who took longer parenting leave, we found no difference in parenting stress. One explanation would be that taking parenting leave might increase fathers’ participation in the household and in childcare, lessening stressors between partners but increase other sources of stress, or it might be another indication that the EBI is not well suited to measuring fathers’ parenting stress, as indicated by our results.

### Limitations

As can be seen at several points in this study, there are various limitations. Recruitment was very broad, but presumably also very selective, such as, for example, a graphic view of education (higher) or parenting status (less single parent) shows. A prospective normalization study should include drawing a representative sample.

An effect of the sample (thesis) was found, with statistically significant differences in values concerning the parental stress experience. Although the studies were designed and structured in a similar way (no test leader present, online or paper-and-pencil possible), this suggests that there is an additional effect of method.

The representativity of the study is certainly also limited due to the very long survey period. Nevertheless, the current study provides valuable information for future research because of its large sample and specificity.

Due to the item–scale correlations, there are also indications that fathers’ parental stress experience could also differ in terms of content from that of mothers’. A further qualitative study could provide information as to whether the reported correlations are due to different aspects included in fathers’ parental stress or maybe just a result of item wording.

The greatest weakness is certainly the exploratory nature of the study, which means that one of the central questions, whether fathers differ in accordance with parental leave, cannot be answered. A future study should investigate this systematically, analyzing the duration of parental leave, as well as comparing the maternal and parental stress experience in a dependent sample.

## 5. Conclusions

In conclusion, as it stands right now, the EBI does not adequately measure parenting stress in fathers, and father specific norms are needed to properly assess their levels of parenting stress. The results concerning parenting stress and parental leave were thus inconclusive. Furthermore, since reducing parenting stress in fathers is beneficial to the child’s development and the welfare of the parents, further studies focusing on fathers’ parenting stress are needed.

## Figures and Tables

**Table 1:** Overview of the three samples and their sociodemographic characteristics.

	Sample 1 (*n* = 39)	Sample 2 (*n* = 249)	Sample 3 (*n* = 208)	Total (*N* = 497)	Reference (*N* = 538)
Nationality					
German	35	238	201	474	220
Non-German	4	11	7	22	25
Single parent					
Yes	2	0	8	10	108
No	38	249	200	487	429
Amount of children					
1	19	140	78	237	220
2	16	78	96	190	240
3	3	20	24	47	55
4 or more	0	11	10	21	22
Education					
low	0	20	4	24	92
middle	8	43	11	62	155
high	31	186	193	410	286
Sex of the youngest child					
girl	18	124	100	242	256
boy	22	125	108	255	282
Parental leave					
Yes	10	143	145	298	
No	12	106	63	181	

Note. The sample sizes differ from 495 to 497, depending on the variable. Regarding readability, missing values are not directly reported.

**Table 2:** Item analysis of the EBI regarding Child Characteristics for Fathers.

Item/Subscale	*M*	*SD*	Corrected Item–Total Correlation	Cronbach’s α Fathers - Mothers
Distractability/Hyperactivity	11.77	14.172		0.665 - 0.75
1	2.92	1.131	0.001	
4	2.91	1.298	0.675	
8	2.99	1.542	0.626	
15	2.95	1.326	0.564	
Mood	11.67	29.375		0.937 - 0.70
34	2.91	1.510	0.815	
44	2.87	1.378	0.838	
45	2.95	1.503	0.884	
48	2.94	1.513	0.872	
Acceptability	11.84	21.465		0.781 - 0.70
19	2.99	1.629	0.764	
22	2.91	1.191	0.166	
28	3.00	1.595	0.691	
30	2.93	1.508	0.775	
Demandingness	11.84	3.389		0.593 - 0.68
38	2.92	1.248	0.277	
39	2.96	1.342	0.247	
43	2.97	1.198	0.502	
47	2.99	1.259	0.501	
Adaptability	11.90	18.310		0.827 - 0.77
17	2.97	1.388	0.750	
20	2.95	1.325	0.715	
26	3.01	1.299	0.554	
31	2.98	1.259	0.599	

Note. The sample sizes differ from 495 to 497, depending on the scale. The reliability of the scales for mothers are based on the manual of the EBI [25].

**Table 3:** Item analysis of the EBI regarding Parent Characteristics for Fathers.

Item/Subscale	*M*	*SD*	Corrected Item–Total Correlation	Cronbach’s α Fathers - Mothers
Parental Attachment	12.03	17.149		0.783 - 0.61
21	2.98	1.323	0.737	
23	2.99	1.206	0.742	
25	3.01	1.263	0.383	
27	3.05	1.510	0.542	
Isolation	11.29	13.532		0.613 - 0.63
7	2.80	1.356	0.576	
9	2.81	1.268	0.152	
11	2.83	1.336	0.503	
13	2.86	1.443	0.376	
Competence	11.87	19.309		0.835 - 0.83
18	3.01	1.267	0.558	
24	2.93	1.452	0.697	
29	2.92	1.312	0.695	
32	3.01	1.333	0.719	
Depression	12.09	12.796		0.576 - 0.75
35	2.98	1.374	−0.078	
37	3.02	1.285	0.603	
40	3.01	1.375	0.542	
42	3.00	1.357	0.513	
Health	11.73	16.270		0.733 - 0.75
2	2.97	1.575	0.349	
6	2.90	1.296	0.657	
10	2.91	1.249	0.537	
12	2.95	1.267	0.607	
Role Restriction	11.64	14.526		0.755 - 0.82
3	2.86	1.286	0.562	
5	2.92	1.255	0.563	
14	2.94	1.276	0.636	
16	2.92	1.200	0.450	
Spouse	11.87	11.216		0.442 - 0.80
33	2.99	1.294	0.387	
36	2.99	1.408	0.123	
41	2.94	1.386	0.328	
46	2.96	1.385	0.183	

Note. The sample sizes differ from 495 to 497, depending on the scale. The reliability of the scales for mothers are based on the manual of the EBI [25].

**Table 4:** Characteristics of the overall score and the subscales of the EBI in Fathers.

Subscale and Overall Score	M	SD	Cronbach’s α Fathers - Mothers
Child Characteristics	58.90	18.559	0.936 - 0.91
Parent Characteristics	82.39	19.633	0.901 - 0.93
Parental Stress Index	141.21	35.445	0.949 - 0.95

Note. The sample sizes differ from 495 to 497, depending on the scale. The reliability of the scales for mothers are based on the manual of the EBI [25].

## Data Availability

Data are archived and available at Open Science Framework: Rüther, JN, & Krüger, N (26 August 2020). Data for research article: Parental stress in fathers. Retrieved from osf.io/8wbax.
